# Supplementary material for: Effects of Dietary Supplementation with High Fiber (Stevia Residue) on the Fecal Flora of Pregnant Sows
Source: Animals (Basel). 2020 Nov 30;10(12):2247. doi: 10.3390/ani10122247 (PMC7761306; doi:10.3390/ani10122247)
Supplement: Supplementary file 1 [file animals-10-02247-s001.pdf]

Supplementary Materials

Table 1. Reproductive performance of pregnant sows.

Group / Project	**Control Group**	**20% Stevia Residue Group**	**30% Stevia Residue Group**	**40% Stevia Residue Group**
Average litter size	13.38	11.67	12.33	13.00
Average live births	11.88	10	11	10.67
Weak Litters Born	2.00	0.63	0.86	1.25

Note: The values with no lowercase-letter superscripts within the same row mean no difference ($p > 0.05$), while different lowercase-letters superscript mean significant differences ($p < 0.05$).
